# Editorial: Metabolic Control of Brain Homeostasis

**DOI:** 10.3389/fnmol.2017.00184

**Published:** 2017-06-09

**Authors:** Detlev Boison, Jochen C. Meier, Susan A. Masino

**Affiliations:** ^1^Robert Stone Dow Chair and Director of Neurobiology Research Legacy Research InstitutePortland, OR, United States; ^2^TU Braunschweig, Cell PhysiologyBraunschweig, Germany; ^3^Life Sciences Center, Neuroscience and Psychology, Trinity CollegeHartford, CT, United States

**Keywords:** ketogenic diet, metabolism, epilepsy, brain cancer, Alzheimer's disease, autism, radiation, RNA editing

Fundamental metabolic processes determine homeostasis of neuronal and glial networks at the molecular, cellular, and systems levels. Metabolic resilience promotes brain health and has the potential to prevent or reverse brain disease. A renewed and increasing interest in the relationship between metabolism and homeostasis is evident across multiple disciplines and has the potential to spawn new insights and therapeutic targets. The striking lack of effective treatments in disorders such as brain cancer, Alzheimer's disease, epilepsy, and autism spectrum disorder (ASD) highlights the limitations of conventional, discipline-specific pharmacological approaches.

For this Research Topic authors were encouraged to submit basic research on homeostasis and excitability as well as metabolic mechanisms associated with neurological diseases and novel treatment approaches based on metabolic and homeostatic interventions. This special issue features several papers examining molecular mechanisms involved in neural network homeostasis and higher brain function. From an evolutionary perspective, “early” metabolites such as adenosine (a structural component of ATP and RNA) and glycine (the simplest amino acid) assumed multiple functions as structural building blocks of cellular systems, as biochemical metabolites involved in energy and carbon homeostasis, as epigenetic modulators, and finally (i.e., an evolutionarily “late” function) as receptor ligands and signaling molecules. Not surprisingly, dysregulation of those key metabolites is implicated in virtually every neurological condition, whereas therapeutic reconstruction of biochemical homeostasis has potential for disease prevention and cure (Boison). A molecular link to basic biochemical mechanisms is further provided in an original research article that ascribes a specific role of the methionine cycle-associated enzyme enolase phosphatase 1 in apoptotic response mechanisms triggered by oxidative stress (Zhang et al.). The link between metabolic homeostasis and neuronal activity is evident in the relationship between ion homeostasis and energy demand of fast neuronal network oscillations associated with higher brain functions—including sensory perception, attentional selection, and memory formation and requiring timed synaptic excitation and inhibition with glutamate and GABA, respectively (Kann et al.). Fast neuronal network oscillations are characterized by high oxygen consumption and significant changes in the cellular redox state, indicating rapid adaptations in glycolysis and oxidative phosphorylation. A second review article highlights the complex role of glycogen and its role in brain energy and particularly synaptic plasticity (Waitt et al.). Exquisite sensitivity to metabolic stress is essential for adaptation and plasticity and confers vulnerability of higher brain functions to injury and disease.

Advances in sequencing technologies led to the discovery of amino acid-recoding RNA editing in many gene transcripts. In this context, recent advances in A-to-I and C-to-U RNA editing are reviewed. This mechanism points to the systemic relevance of the neurotransmitter receptor for glycine (GlyR) and possible clues to disease mechanisms. C-to-U RNA editing of GlyR-coding transcripts is increased in the hippocampus of patients with intractable temporal lobe epilepsy and can provoke completely different symptoms depending on the neuron type that is affected (Winkelmann et al., 2014; Çalişkan et al., 2016). These recent discoveries provide further evidence for the concept of excitatory/inhibitory “imbalance” in current psychiatric neuroscience (Eichler and Meier, 2008), but we ultimately need to identify susceptible neuron types and, in particular, differentiate among “inhibitory” GABAergic neuron types (Lovett-Barron et al., 2014; Çalişkan et al., 2016; Meier et al.). An excitatory/inhibitory “imbalance” and excess excitability can have many causes: Westmark et al. delineate the homeostatic role of amyloid-β protein precursor (APP) and its metabolites in fragile X syndrome in humans and in the Fmr1KO mouse model. APP seems to serve as a rheostat where too much or too little causes hyperexcitability—suggesting that normalizing APP levels can address aspects of this pathophysiology.

Regarding translational and clinical research, a study of the healthy human brain indicates the existence of specific metabolomic profiles in different brain regions (Cabré et al.). An analysis of post-traumatic brain injury cerebrospinal fluid finds that mortality at 6 months can be predicted by levels of cortisol and BDNF, particularly in younger people, and suggests a regulatory role for cortisol (Munoz et al.). Several articles look at changes mobilized by a metabolic therapy used to treat epileptic seizures for nearly 100 years: the ketogenic diet (Masino, 2017). These studies include strategies to mimic the diet *in vitro* and in transitional *in vitro*/*in vivo* models (Kawamura et al.). In an original research paper, anxiolytic behavioral effects of ketone-based metabolism—mobilized by administering a ketone ester to induce nutritional ketosis—are reported in two rat strains (Ari et al.). A second original paper compared ketosis induced by exogenous ketones vs. a ketogenic diet and found similar biochemical changes but more robust behavioral effects of the diet (Brownlow et al.). Laboratory and clinical evidence is reviewed regarding the prevalence of dysfunctional mitochondria and altered metabolism in ASD alongside current limited but positive data on the role of ketogenic diets and metabolic therapy in reducing ASD symptoms and common comorbidities—potentially via adenosine or other mechanisms (Cheng et al.). An original research article demonstrates that enhancing adenosine in brain through pharmacological blockade of the enzyme adenosine kinase prevents radiation-induced cognitive impairment in rats (Acharya et al.).

Finally, the role of metabolic factors and the promise of metabolic therapy in brain cancer and Alzheimer's disease are implicated directly in several articles: an original research article reveals a critical role of the proneural basic helix-loop-helix transcription factor human achaete-scute homolog 1 (hASH1) in neuroblastoma. This study shows that hASH1 suppresses neuronal differentiation by inhibiting transcription at the retinoic acid receptor element, highlighting hASH1 as a key determinant of neuroblastoma resistance to differentiation therapy (Kasim et al.). Metabolic therapy with a ketogenic diet is highlighted as a multifaceted therapy for glioma, particularly glioblastoma, and clinical and basic research is reviewed indicating that a metabolic approach may both limit tumor growth and augment the efficacy of chemotherapy and radiation (Woolf et al.). Finally, hypometabolism is a hallmark of Alzheimer's disease, and regional glucose hypometabolism is also found in patients at risk for Alzheimer's disease and may be a presymptomatic marker. However, hypometabolism is reversed by administering ketones as an alternative fuel—suggesting that a selective problem with glucose metabolism may underlie the pathophysiology of early onset Alzheimer's disease, and that a metabolic therapy may prevent or delay the disorder (Cunnane et al.).

Taken together, this research topic provides a cross-cutting perspective of the relationship between metabolism and homeostasis and showcases novel metabolic therapies to restore molecular and biochemical networks. The direct and potentially imminent clinical implications of several of these articles will be exciting to follow as the field moves forward. While a range of topics and mechanisms is featured herein, there is room for much more.

## Author contributions

All authors listed, have made substantial, direct and intellectual contribution to the work, and approved it for publication.

### Conflict of interest statement

The authors declare that the research was conducted in the absence of any commercial or financial relationships that could be construed as a potential conflict of interest.
